# Two small-molecule activators share similar effector sites in the KCNQ1 channel pore but have distinct effects on voltage sensor movements

**DOI:** 10.3389/fphys.2022.903050

**Published:** 2022-07-25

**Authors:** Lei Chen, Gary Peng, Thomas W. Comollo, Xinle Zou, Kevin J. Sampson, H. Peter Larsson, Robert S. Kass

**Affiliations:** ^1^ Department of Molecular Pharmacology and Therapeutics, Vagelos College of Physicians & Surgeons of Columbia University Irving Medical Center, New York, NY, United States; ^2^ Department of Physiology and Biophysics, Miller School of Medicine, University of Miami, Miami, FL, United States

**Keywords:** KCNQ1, channel, agonists, voltage sensor, long QT syndrome

## Abstract

ML277 and R-L3 are two small-molecule activators of KCNQ1, the pore-forming subunit of the slowly activating potassium channel I_Ks_. KCNQ1 loss-of-function mutations prolong cardiac action potential duration and are associated with long QT syndrome, which predispose patients to lethal ventricular arrhythmia. ML277 and R-L3 enhance KCNQ1 current amplitude and slow deactivation. However, the presence of KCNE1, an auxiliary subunit of I_Ks_ channels, renders the channel insensitive to both activators. We found that ML277 effects are dependent on several residues in the KCNQ1 pore domain. Some of these residues are also necessary for R-L3 effects. These residues form a putative hydrophobic pocket located between two adjacent KCNQ1 subunits, where KCNE1 subunits are thought to dwell, thus providing an explanation for how KCNE1 renders the I_Ks_ channel insensitive to these activators. Our experiments showed that the effect of R-L3 on voltage sensor movement during channel deactivation was much more prominent than that of ML277. Simulations using a KCNQ1 kinetic model showed that the effects of ML277 and R-L3 could be reproduced through two different effects on channel gating: ML277 enhances KCNQ1 channel function through a pore-dependent and voltage sensor–independent mechanism, while R-L3 affects both channel pore and voltage sensor.

## Introduction

In excitable cells such as cardiac myocytes and neurons, potassium channels play critical roles in controlling cell excitability (Nerbonne and Kass, 2005). They set the resting membrane potential close to the potassium equilibrium potential; determine the duration, shape, and the firing frequencies of action potentials; and prevent cells from being overly excited. Loss-of-function of potassium channels leads to prolonged cellular repolarization and hyperexcitability and may result in diseases such as epilepsy and cardiac arrhythmia. Potassium channel activators promote channel opening and may be capable of restoring normal physiological properties of the dysfunctional channels. These pharmacological agents thus provide potential therapeutic means for a wide range of diseases involving cellular hyperexcitability. However, the great diversity of potassium channels, which is manifested by their distinct molecular identities, auxiliary subunit compositions, electrophysiology characteristics, and tissue specific expressions, poses a challenging task for pharmacologists to identify potent yet specific activators (Wulff et al., 2009).

The I_Ks_ channel is a slowly activated voltage-gated potassium channel that plays a critical role in cardiac myocytes. In response to membrane depolarization, these channels open slowly during the plateau phase of cardiac action potentials and help return membrane potential back to the resting level. Working in concert with other voltage-gated potassium channels, the I_Ks_ channel is a critical player in cardiac repolarization and a major determinant of the ventricular action potential duration (APD). Reduction of I_Ks_ channel activity prolongs the APD and may predispose cardiac myocytes to abnormal electrical activity such as early after depolarizations (EADs) that are capable of triggering lethal cardiac arrhythmia. This clinical condition is observed as the long QT syndrome (LQTS) since the abnormally long intervals between the QRS complex and T wave on a patient’s ECG are a manifestation of prolonged cardiac ventricular APDs (Keating and Sanguinetti, 2001). The K^+^ ion conducting α-subunit of I_Ks_ channel is KCNQ1. It is, however, the auxiliary β-subunit KCNE1 that determines the characteristic slow activation kinetics of the I_Ks_ channels (Barhanin et al., 1996; Sanguinetti et al., 1996). Naturally occurring loss-of-function mutations in both KCNQ1 and KCNE1 have been identified to cause congenital LQT1 (Wang et al., 1996) and LQT5, respectively (Splawski et al., 1997).

Because of their potential therapeutic utilities in treating cardiac arrhythmias, activators of I_Ks_ channels have been long sought after. Several synthetic small-molecule compounds are known to enhance KCNQ1 or KCNQ1/KCNE1 channel functions (Xiong et al., 2008): DIDS and N-phenylanthranilic acid derivatives, such as mefenamic acid, tend to promote KCNQ1/KCNE1 activity (Busch et al., 1997), whereas zinc pyrithione preferentially activates KCNQ1 in the absence of KCNE1 (Xiong et al., 2007). Another example of the later is ML277 identified by high-throughput screening. ML277 activates KCNQ1 in a sub-micromolar range (Yu, 2010). It increases current amplitude and slows channel deactivation kinetics. It is selective against other cardiac ion channels such as Na_V_1.5, hERG, and Ca_V_1.2 as well as other members in the Kv7.x family, such as KCNQ2 and KCNQ4 (Mattmann et al., 2012). R-L3 is another KCNQ1 activator in the micro-molar range, which enhances channel activity in a similar fashion as ML277 (Salata et al., 1998; Maljevic et al., 2010). Most interestingly, both ML277 and R-L3 show a strong preference for channels composed of KCNQ1 only, without its auxiliary KCNE1 subunits. As the stoichiometry between KCNQ1 and KCNE1 approaches 1:1, the functional channels lose their sensitivity to both small-molecule activators (Xiong et al., 2008; Yu et al., 2013).

To understand the mechanisms underlying how ML277 and R-L3 activate the channel, we sought to identify, in the KCNQ1 channel, key domains and residues critical for drug effects. Using a combination of mutagenesis, voltage-clamped current and fluorescence recordings, and channel kinetics modeling, we now report that ML277 and R-L3 share common action sites in a hydrophobic pocket in the KCNQ1 S5–S6 pore domain. While ML277 activates the KCNQ1 channel through a pore-dependent, voltage sensor–independent mechanism, R-L3 has a potent effect on voltage sensor deactivation.

## Materials and methods

### Constructs and mutagenesis

Wild-type KCNQ1 was cloned in the pGEMHE vector. Wild type KCNQ2 and KCNQ1/2 chimeras were gifts from Drs. Guiscard Seebohm and Michael Sanguinetti (Maljevic et al., 2003; Seebohm et al., 2005). Sequence alignment was performed using CLC Sequence Viewer 7.0.2 (CLCbio, a Qiagen Company, Boston, MA). Mutations of KCNQ1 were created using the QuikChange site-directed mutagenesis kit (Agilent Technologies, Santa Clara, CA) according to the manual. Sequences of the entire open reading frame of the mutated KCNQ1 were confirmed.

### cRNA preparations and *Xenopus* oocyte injection

cRNAs were prepared by first linearizing the DNA plasmid using NheI-HF, which is followed by *in vitro* RNA transcription using the mMESSAGE mMACHINE T7 kit (Ambion, Austin, TX). The concentration of cRNA was determined by measuring absorbance at 260 nm. Xenopus oocytes were purchased from Ecocyte Bioscience (Austin, TX). cRNAs (1 ng/nL) for KCNQ1, and mutants were injected into oocytes (41.2 nL per oocyte) using a Drummond injector (Broomall, PA). Oocytes were incubated at 16°C for ∼36–48 h before recordings.

### Two electrode voltage clamp recordings

Oocytes were placed in a recording chamber filled and perfused with ND96 solution (96 mM NaCl, 2 mM KCl, 5 mM HEPEs, 1.8 mM CaCl2, 1mM MgCl2, and pH 7.5). A total of 100 uM LaCl3 was used to block the endogenous currents in the oocytes. The oocytes were held at −80 mV. KCNQ1 currents were evoked by depolarizing the oocytes to +20 mV for 2 s and then repolarizing to 120 mV for 2 s and were recorded every 15 s to monitor the drug effect. To record the activation curve for the KCNQ1 channel, oocytes were first subject to testing pulses from −140 to +80 mV (in 20-mV increments) for 2 s, and then, they were subject to a 2 s −120 mV pulse. Current amplitudes were measured isochronally at the end of the depolarization pulse. The time constant τ was obtained by fitting the tail current using single exponential equation to measure the channel deactivation kinetics. For some chimera channels, it was not possible to fit the tail current using this method. In such cases, the half time (t1/2) of tail current decay was used to measure deactivation kinetics. Channel activation curves were fitted using the Boltzmann equation.

### Voltage clamp fluorometry recordings

Fifty nano grams of KCNQ1 RNA were injected into defoliculated *Xenopus laevis* oocytes. Experiments were performed two to four days after injection. Oocytes were labeled for 30 min with 100 µM Alexa 488-maleimide (Molecular Probes) in regular ND96 (96 mM NaCl, 2mM KCl, 1.8 mM CaCl_2_, 1mM MgCl_2_, and 5 mM HEPES (pH 7.5) with NaOH). Following labeling, oocytes were kept over ice to prevent internalization of labeled channels. Oocytes were placed into a recording chamber in ND96. A volume of 100 µM LaCl_3_ is used to block endogenous hyperpolarization-activated currents. VCF experiments were carried out as previously reported (Osteen et al., 2010; Osteen et al., 2012).

### Kinetic modeling

A Markov model of channel gating was used to analyze and interpret the VCF and current recordings. This model has 12 states, corresponding to closed and open states for four independent voltage sensing domains with a final concerted opening step as published previously (Peng et al., 2017). Simulations were performed using MATLAB (Natick, MA).

### Flow cytometry

The plasmid BBS-KCNQ1-YFP was a generous gift from Dr. Henry M Colecraft (Physiology & Cellular Biophysics, Columbia University). Cell surface experiments and total ion channel pools were assayed using flow cytometry in live, transfected HEK293 cells as previously described (Kanner et al., 2017; Kanner et al., 2018; Kanner et al., 2020). Briefly, 48 h post-transfection, cells cultured in six-well plates were gently washed twice with 1 ml ice cold PBS containing Ca^2+^ and Mg^2+^ (in mM: 0.9 CaCl_2_, 0.49 MgCl_2_, and pH 7.4), and then, the cells were incubated for 30 min in 1 ml blocking medium (DMEM with 3% BSA) at 4 _°_C. HEK293 cells were then incubated with 0.5 ml 1 mM Alexa Fluor 647 conjugated α-bungarotoxin (BTX_647_; Life Technologies) in blocking medium at 4 _°_C for 1 h on a rocker, followed by washing three times for 5 min with PBS (containing Ca^2+^ and Mg^2+^). Cells were gently harvested in Ca^2+^ -free PBS, and they were assayed by flow cytometry using the BD LSRII Cell Analyzer (BD Biosciences, San Jose, CA, United States). YFP-tagged proteins were excited at 488 nm, and Alexa Fluor 647 was excited at 633 nm. For the ML277 or R-L3 treatment groups, cells were pre-treated with 1 μM ML277 or R-L3, respectively, for 10 min prior to the experiments. To balance the control data, we prepared two equal control groups. Cells were placed at 4 _°_C to halt trafficking processes and were washed twice with PBS containing Ca^2+^ and Mg^2+^.

## Results

**FIGURE 1:**
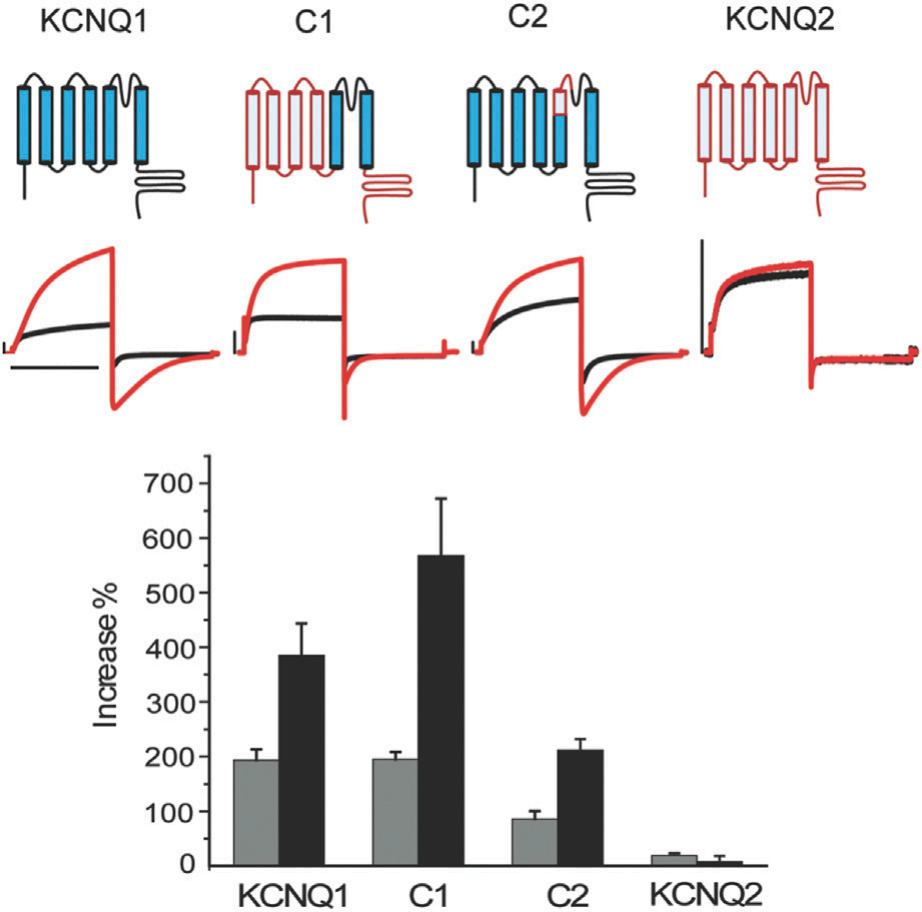
KCNQ1 pore domain is the main drug site for ML277. Top panel) schematic structure of wt KCNQ1 (blue), WT KCNQ2 (red), and chimera channels C1 and C2. C1 consists of KCNQ1 S5-pore-S6 and KCNQ2 S1-4 and N, C-termini. C2 is mostly KCNQ1 except for the top of S5 and a small portion of the pore loop that was replaced by the KCNQ2 counterparts. Middle panel) representative current traces recorded at control condition (black) and with 10 uM ML277 (red). Bottom panel) summary of the effects of ML277 on current amplitude (grey bar) and deactivation kinetics as measured by t1/2 of tail current decay (black bar). The scale bar in the figure is a 1 s time bar.

**FIGURE 2:**
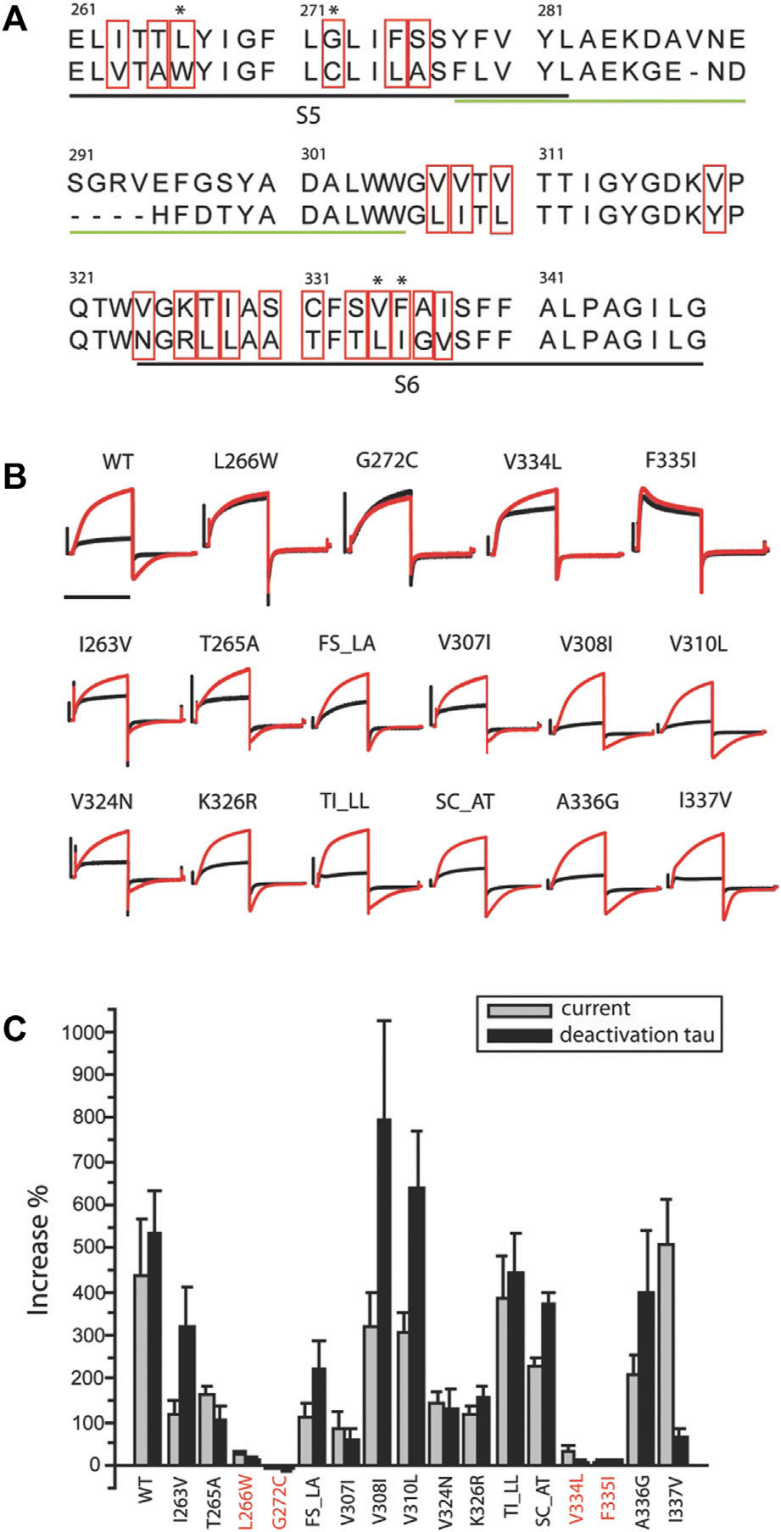
Residues critical for ML277 binding. **(A)** Sequence alignment of KCNQ1 and KCNQ2 pore domains. The black underlined residues are located in the S5 and S6 transmembrane domains. The green underlined residues were excluded by chimera C2 and are unlikely drug sites for ML277. The residues in red boxes are not conserved between KCNQ1 and KCNQ2 and are potential sites critical for ML277 binding. **(B)** KCNQ1 residues L266, G272, V334, and F335 are responsible for ML277 effects. Current traces in the top row show the effect of ML277 on wt and the loss of effect on L266W, G272C, V334L, and F335I KCNQ1. The middle and lower rows show the effect of ML277 on other mutant channels. The black and red traces represent recording in control condition and with 10uM ML277, respectively. **(C)** Bar graph summarizing the effect of ML277 on current amplitude (grey bar) and deactivation kinetics (black bar) of all the mutants tested. The scale bar in panel B is a 1 s time bar.

ML277 selectively enhances the activities of KCNQ1, but not the closely related KCNQ2. Both KCNQ1 and KCNQ2 belong to the Kv7 family and share similarity in the primary sequences, especially in the transmembrane domains. We thus adopted an approach using KCNQ1 and KCNQ2 chimeric channels to locate potential site(s) critical for ML277 function. ML277 causes an increase in KCNQ1 current amplitude and a slowing of deactivation kinetics as measured by the time constant or t_1/2_ of the tail current decay. As shown in Figure 1, the current amplitudes of WT KCNQ1 channels were increased by 194 ± 19% (n = 5) and the time constant for deactivation kinetics was increased by 385 ± 58% upon the application of 10 uM ML277. Consistent with previous reports, the effects of ML277 on KCNQ2 were minimal with a 19 ± 4% increase in current amplitude and a 7 ± 10% increase in deactivation t_1/2_ (n = 5) (Figure 1). Chimera C1 possesses the KCNQ1 pore (S5-P-S6) and KCNQ2 N-, C-termini and voltage sensing domain (S1–S4) (Seebohm et al., 2003a). ML277 increased C1 current amplitude by 195 ± 14% and slowed deactivation t_1/2_ by 567 ± 105% (n = 5) (Figure 1). This suggests that KCNQ1 pore domain (S5-P-S6) may contain site(s) necessary for ML277 actions. Chimera C2 is mostly KCNQ1 except for a small portion at the top of S5 as well as part of the P-loop that was replaced by the corresponding sequences of KCNQ2 (green underlined sequence in Figure 2) (Seebohm et al., 2003a). ML277 increased C2 current amplitude by 86 ± 15% and slowed deactivation t_1/2_ by 212 ± 20% (n = 5). This suggests that the top of the KCNQ1 S5 and the small part of P-loop that connects to the S5 are not necessary for ML277 actions. We also tried other chimeric channels with different parts of KCNQ1 and KCNQ2. None yielded favorable expression levels to reliably assay ML277 effects.

Based on these findings, we conclude that the KCNQ1 voltage sensor is unlikely the target of ML277 and that the primary site required for ML277 effect is confined within the S5–S6 pore domain. Next, we compared the amino acid sequences of KCNQ1 and KCNQ2 pores. Figure 2A shows an alignment of the S5-P-S6 domains of KCNQ1 and KCNQ2, which was implicated by chimera C1 to be necessary for ML277 actions. The small region on the top of S5 and nearby P-loop is probably not necessary for ML277 effects, as suggested by the results of chimera C2 shown in Figure 1. KCNQ1 residues that are different from KCNQ2 are potentially important for ML277 effects and are shown in red boxes in Figure 2A. We then mutated these residues one or two at a time to the corresponding ones on KCNQ2 for functional examination. It should be noted that WT data in Figures 1,2 are from two sets of experiments. Each figure is an individual set of experiments for which data from WT cells were included as positive controls. That is why the drug responses for WT records in Figures 1, 2 are different in magnitude but follow the same trends in both figures. The same experimental conditions were used in these experiments, but this approach was used to take into account natural cell and/or channel expression variability in the experiments.

All but two mutants (V319Y and S333T) expressed well in oocytes. Figure 2B shows recordings of all 16 expressed mutants and WT. The double mutants that are illustrated (TI_LL and SC_AT) did not affect drug actions, and thus allowed us to eliminate single mutations of the residues T, I, S, and C. Four mutants (L266W, G272C, V334L, and F335I) showed minimal to no response to ML277 as measured by increases in current and deactivation kinetics. Unlike WT KCNQ1 that responded to ML277 by an increase in current amplitude by 437 ± 132% and an increase in deactivation time constant (τ) by 533 ± 97% (n = 9), four mutations drastically decreased the effect of ML277. L266W showed increases of only 26 ± 8% and 14 ± 5% (n = 5) in current amplitude and deactivation τ, respectively; G272C showed a change of −4 ± 3% and −4 ± 9% (n = 5) in current amplitude and deactivation τ, respectively; V334L showed an increase of 36 ± 8% and 5 ± 5% (n = 5) in current amplitude and deactivation τ, respectively; and F335I showed an increase of 5 ± 5% and 12 ± 3% (n = 5) in current amplitude and deactivation τ, respectively. All other mutants responded robustly to ML277. Results are summarized in the bar graph in Figure 2C. Some mutants produced a preferential response to ML277 in either current amplitude or deactivation kinetics. For example, I337V showed a very robust increase in current amplitude (511 ± 99%), but a somewhat muted response in deactivation kinetics (67 ± 17%), an effect much smaller than that of WT (533 ± 97%). These results demonstrated that mutations of four residues (L266, G272, V334, and F335) in the KCNQ1 pore domain greatly diminished the effects of ML277 on current amplitudes and deactivation kinetics. These residues are critical for the functional effect of ML277.

**FIGURE 3:**
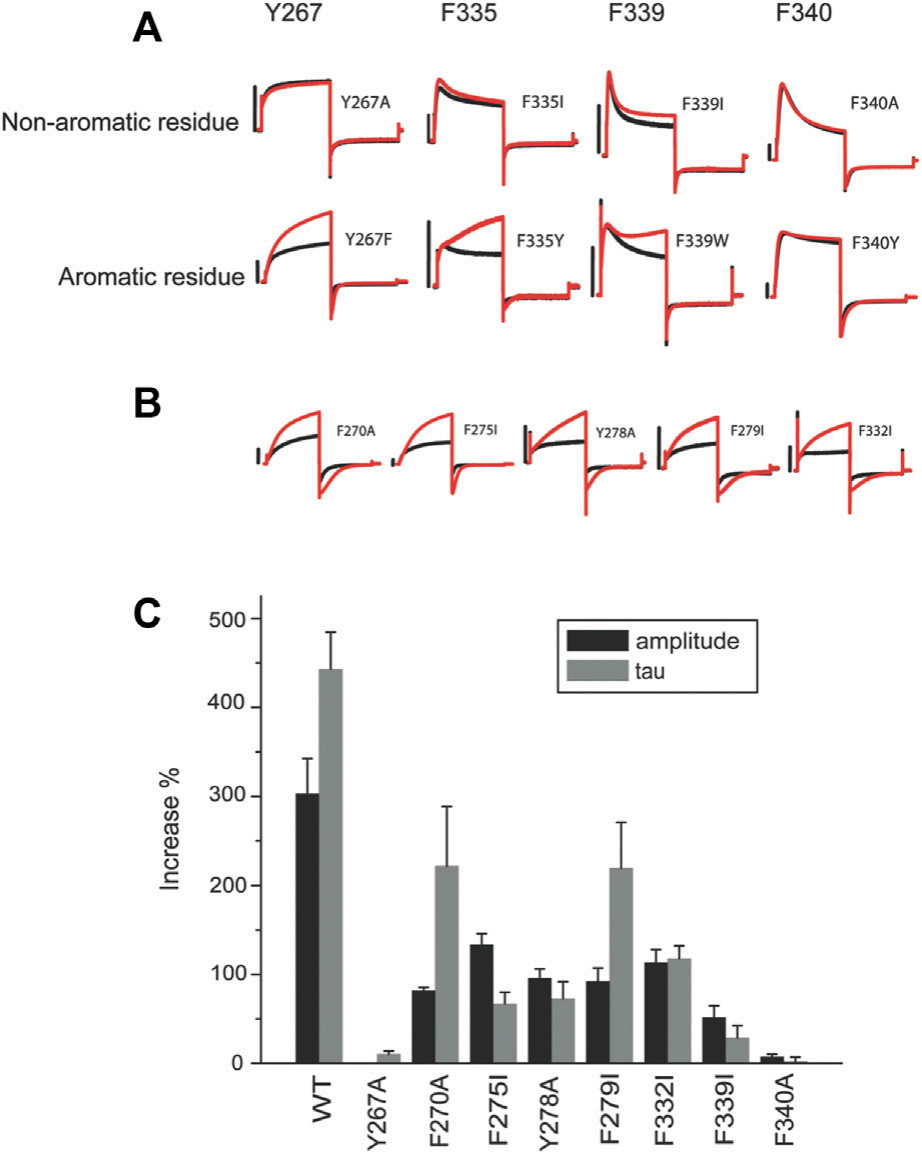
Aromatic residues in a putative hydrophobic pocket are important for ML277 activity. **(A)** Y267, F335, and F340 are required for ML277 effect. Representative recordings of KCNQ1 Y267, F335, F339, and F340 mutated to non-aromatic residues (top panel) and aromatic residues (lower panels) before (black) and after (red) application of ML277. **(B)** Representative recordings of other aromatic residues in KCNQ1 mutated to non-aromatic residues before (black) and after (red) application of ML277. **(C)** Bar graph summarizing the effect of ML277 on current amplitude (grey bar) and deactivation kinetics (black bar) of all the mutants tested. The scale bar in the figure is a 1 s time bar.

**FIGURE 4:**
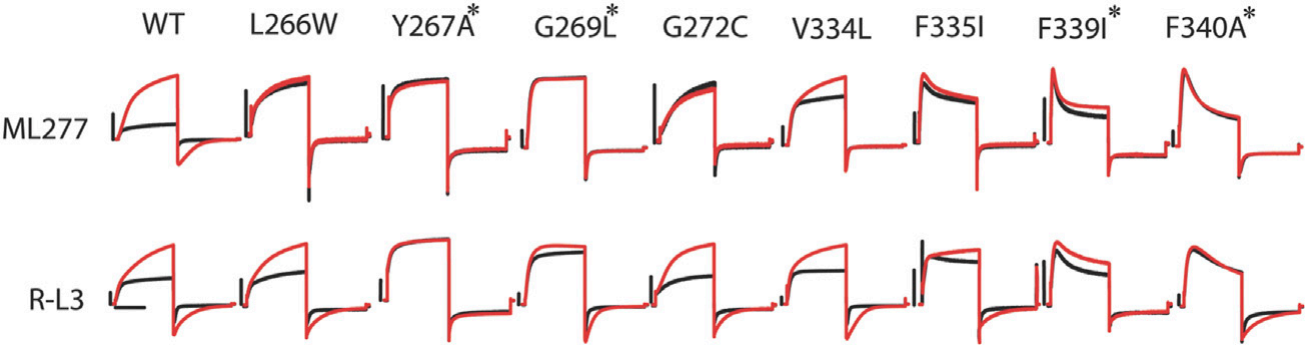
R-L3 shares some common residues with ML277 for functional effects. Representative recordings of mutants of all eight residues necessary for ML277 effect before (black) and after (red) ML277 application (top panel). Representative recording of the same mutants before (black) and after (red) R-L3 application (lower panel). Common residues that abolish the effects of both ML277 and R-L3 are marked with a star (*). The scale bar in the figure is a 0.5 s time bar.

While revealing the required residues, the mutagenesis study shown in Figure 2 did not necessarily suggest that these four residues are sufficient for ML277 effect. Aromatic residues in the channel pore have long been recognized as critical for drug binding and could serve as additional sites for ML277 interaction. The small size of the glycine residue (G269), on the other hand, may provide accommodative environment for drug binding. We next tested these two possibilities. We first mutated all aromatic residues (phenylalanine, tryptophan, and tyrosine) to non-aromatic ones (Figure 3). Compared with WT KCNQ1 and other mutants, six mutants (Y267A, F335I, F339I, F339W, F340Y, and F340A) showed a loss of sensitivity to ML277 to various degrees (increase of current amplitude: wt 303 ± 39%, Y267A 1 ± 2%, F339I 52 ± 13%, and F340A 8 ± 3%; increase of τ: wt 443 ± 41%, Y267A 10 ± 4%, F339I 29 ± 14%, and F340A 2 ± 5%; and n = 12 for WT, n = 5 for Y267A and F340A, and n = 6 for F339I) (Figure 3A top, results of F335I are shown in Figure 2), whereas mutations of five other KCNQ1 aromatic residues still maintained ML277 effects (Figure 3B). Interestingly, upon mutating to a different aromatic residue, for example, from phenylalanine to tyrosine or vice versa, Y267F and F335Y partially maintained channel’s sensitivity to ML277, especially in terms of current increase (increase of amplitude: Y267F 114 ± 15%, n = 5; F335Y 107 ± 28%, n = 8) (Figure 3A bottom). This suggests that the aromatic rings may be critically involved in drug binding. Next, we tested the hypothesis whether mutating G269 to a residue with a larger side chain may prevent ML277 from binding to the proposed hydrophobic pocket. Indeed, we found that G269L KCNQ1 no longer responded to ML277 (amplitude increase: 3 ± 0%, increase of τ 2 ± 3%, n = 6, Figure 4 upper row).

Given the functional similarity between the effects of ML277 and R-L3 on channel currents, we speculated that R-L3 may share some common residues with ML277 to affect the KCNQ1 channel. In Figure 4, we show that four mutations (Y267A, G269L, F339I, and F340A) that made the KCNQ1 channel insensitive to ML277 also rendered the channel at least partially irresponsive to R-L3. Among them, Y267A abolished the effect of R-L3 on both current amplitude and deactivation kinetics (increases in current amplitude and deactivation τ in wt KCNQ1 were 119 ± 8% and 542 ± 75%, respectively, n = 9; and increases in current amplitude and deactivation τ in Y267A were 1 ± 1% and −5 ± 5%, respectively, n = 5). The effect of G269L, F339I, and F340A was limited only to abolishing the R-L3-induced increase in current amplitude (increases in current amplitude in G269L, F339I, and F340A were 12 ± 5%, 23 ± 5%, and −4 ± 4%, respectively, n = 5). G269L, F339I, and F340A KCNQ1 remained responsive to R-L3 in terms of deactivation kinetics (increases in deactivation τ for G269L, F339I, and F340A KCNQ1 were 216 ± 33%, 111 ± 6%, and 298 ± 25%, respectively, n = 5). These results are suggestive of a separation in mechanisms underlying current amplitude increase and changes in deactivation kinetics caused by R-L3 and are in sharp contrast of ML277, where all identified mutations abolished their effect on both current amplitude and deactivation kinetics.

**FIGURE 5:**
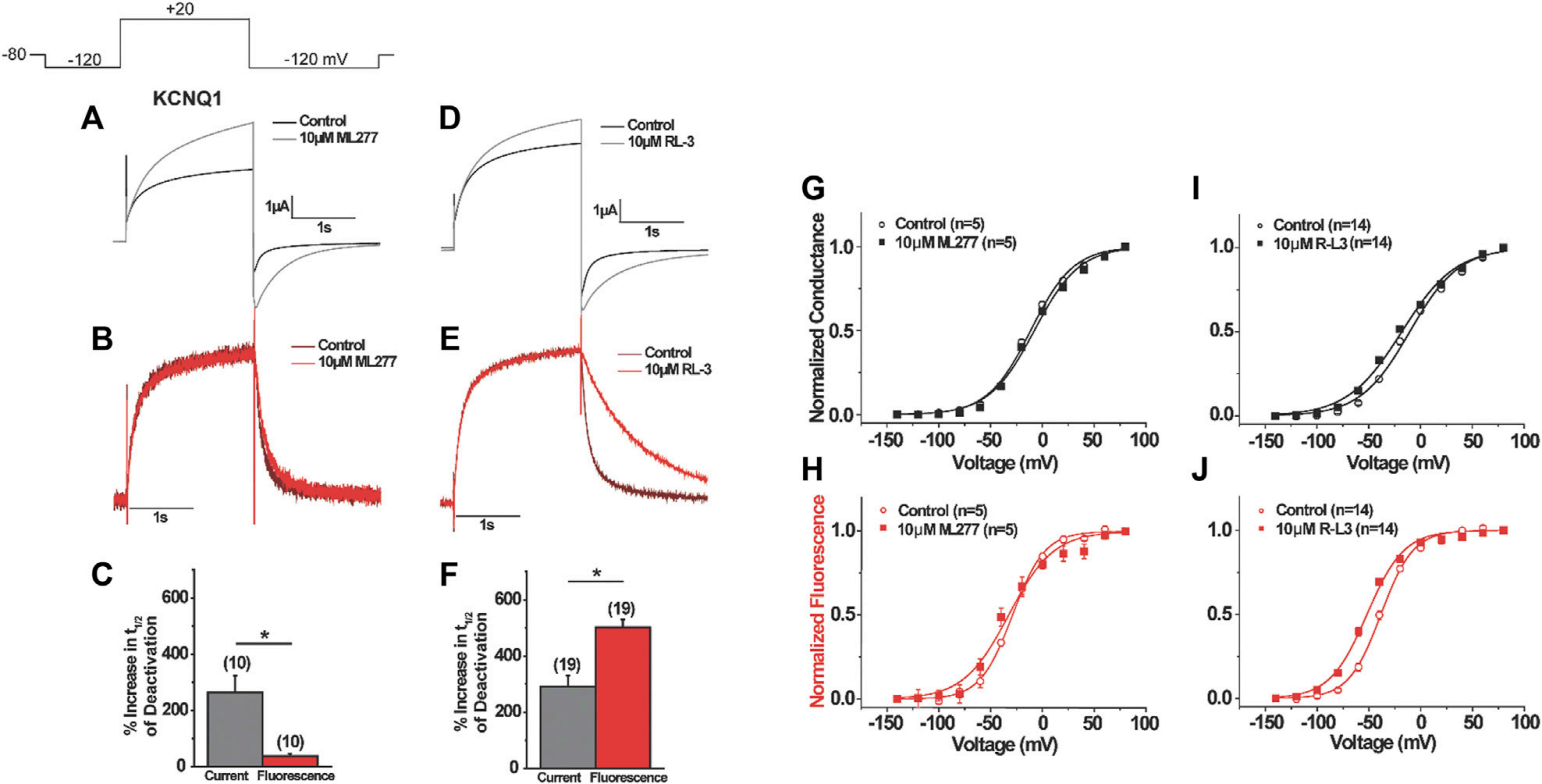
Voltage clamp fluorometry of the effects of ML277 and RL-3 on KCNQ1. **(A–F)** Representative current (top) and fluorescence (middle) recordings of KCNQ1 before and after **(A,B)** ML277 application or **(D–E)** R-L3 application. Voltage protocol shown in inset. **(C,F)** Summary of **(C) **ML277 and **(F) **R-L3 effects on the deactivation of the current (gray) and fluorescence (red). **(G–J)** G(V) (top) and F(V) (bottom) for KCNQ1 before (open symbols) and after (closed symbols) application of ML277 **(G–H)** or R-L3 **(I–J)**.

Based on the mutant KCNQ1 behavior in response to ML277 and R-L3, we speculate that the two molecules may have distinct effects on KCNQ1 voltage sensors. We then measured the voltage sensor movements in the presence of ML277 and R-L3. Using voltage clamp fluorometry (VCF), which allows for simultaneous detection of voltage sensor movement and current conduction, we first explored changes in channel voltage sensor movements as a result of ML277 application. We used a pseudo-wild type (psWT) KCNQ1 construct containing two neutralized cysteine residues (C214A/C331A) to minimize background signals and an introduced cysteine (G219C) at the S3–S4 linker to be cross-linked to a fluorophore in order to monitor conformational changes associated with voltage sensor movement as previously described (Osteen et al., 2010). Like true wild type KCNQ1, we found that the application of 10 µM ML277 significantly increased the current amplitude of the psWT KCNQ1 by 95 ± 8%. ML277 also slowed current deactivation recorded at −120 mV, with a 289% increase in the deactivation time constant τ (Figure 5A). In simultaneous current and fluorescence recordings using VCF, ML277 did not change the activation kinetics of the fluorescence, but mildly slowed deactivation kinetics of the fluorescence, with an 88% increase in fluorescence deactivation time constant (Figure 5B), an effect much smaller than that of the current (289%). We also studied the effects of ML277 on the voltage dependence of activation of psWT KCNQ1 current, G(V), and voltage sensor movement, F(V), and did not observe any significant changes in the V_1/2_ of the isochronal G(V) or F(V) (Figure 5G-H). These results suggest that ML277 has a minimal impact on the voltage sensor movement and likely affects the pore domain of the KCNQ1 channel.

**FIGURE 6:**
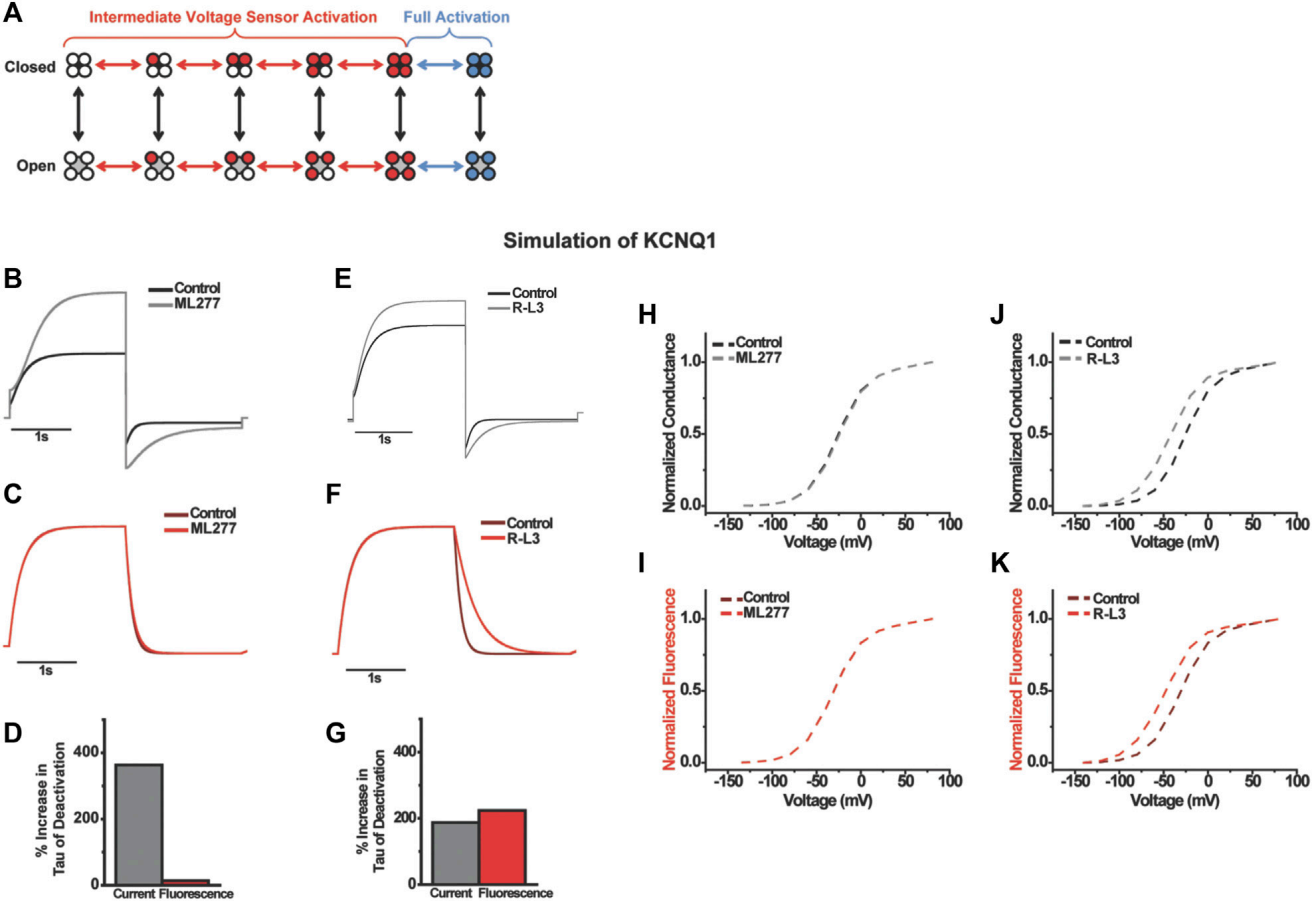
Computational modeling the effects of ML277 and RL-3 on KCNQ1. A 12-state model with intermediate and activated states was used **(A)**. ML277 was modeled as a pure modulator slowing pore opening with no effect on voltage sensing while RL-3 was modeled with only changes to deactivation of voltage sensing. Current, fluorescence, and time to half inactivation agree with experiments **(B–G)** and the voltage-dependent properties **(H–K)**.

To investigate the effects of R-L3 on the voltage-sensing domain, we also used VCF to record voltage sensor movement and current simultaneously. We found that R-L3 significantly increased the current amplitude of pseudo–WT KCNQ1 by 28 ± 4% and the time constant of current deactivation by 199 ± 22% (Figure 5D). In contrast with ML277, which showed a minor change in fluorescence deactivation constant by 88% and no shift in G(V) and F(V), R-L3 slows fluorescence deactivation more prominently, with a 335 ± 22% increase in the fluorescence deactivation time constant (Figures 6E,F). In addition, R-L3 causes a significant hyperpolarizing shift in the G(V) by −6.0 ± 2.0 mV and in the F(V) by −13.8 ± 2.1 mV (Figures 6I,J).

**FIGURE 7:**
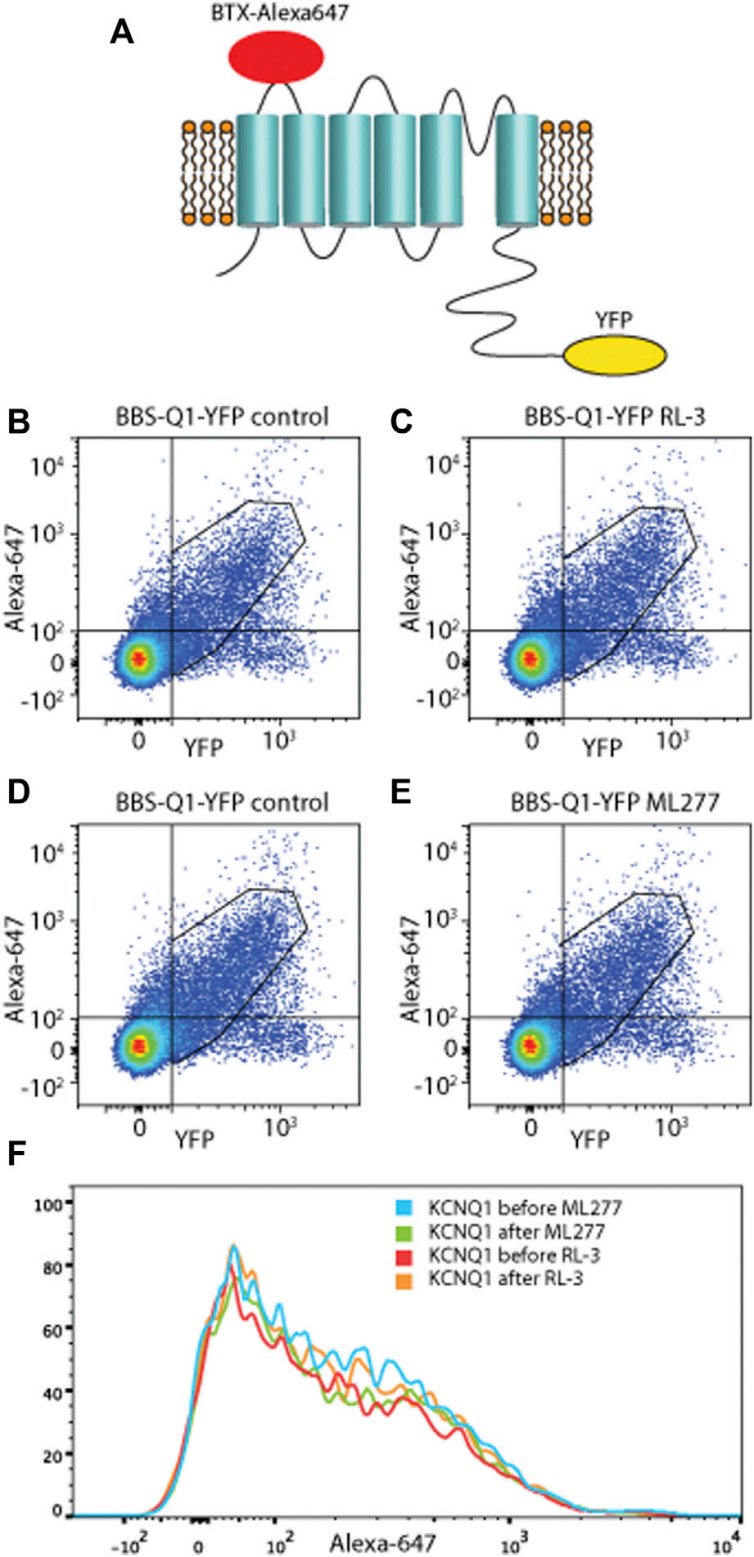
ML277 and R-L3 do not alter membrane expression. Scatter plots from Bungarotoxin (BTX_647_) flow cytometry experiments showing the detection of BTX_647_ (extracellular marker) and YFP (intracellular tag) where dots in the upper right quadrant represent channels that expressed and trafficked to the membrane. **(A)** Schematic diagram of KCNQ1 with BTX-Alexa 647 and YFP. For both RL-3 **(B,C)** and ML277 **(D,E)**, there was no change in surface expression of channels between control condition (left panels) and after application of drug (right panels). **(F)** Shows the data as compressed onto the *y*-axis to further emphasize no significant change.

To understand the differences in gating effects between ML277 and R-L3 on the KCNQ1 channel, we turned to kinetic modeling to simulate the drug effects (Figure 6). Could the mild effect of ML277 on voltage sensor movement during channel deactivation be an indirect effect occurring by retrograde coupling through the pore, and does R-L3 use a different mechanism compared to ML277 to directly alter voltage sensor movements? We modeled the effects of ML277 and R-L3 on channel gating using the same gating scheme developed in previous studies (Peng et al., 2017). In this scheme, which is illustrated in Figure 6A, voltage sensor movement is represented by the horizontal transitions and occurs in two steps: first, independent movement of the four S4s to an intermediate state; second, a concerted step of all four S4s to the fully activated state. Previous studies of KCNQ1 show that channel opening occurs mainly when some or all voltage sensors are in the intermediate state (Zaydman et al., 2014). Each step in voltage sensor activation increases the probability of channel opening in an allosteric manner (Osteen et al., 2012). To simulate the gating effect of ML277, we slowed the pore opening and closing transitions in addition to increasing the current amplitude from our KCNQ1 model (Supplementary Table S1). We simulated current and fluorescence using the same single-pulse voltage protocol as in our experiments (Figures 6B–F). In addition, we simulated F(V) and G(V) curves. Our simulation shows slowing in deactivation and slight slowing in activation (Figure 6B), similar to the effects of ML277. The time constant of current deactivation is increased by 363% and accompanied by a small (14%) increase in the time constant of fluorescence deactivation (Figures 6B–D). Furthermore, there is no change in the activation kinetics of fluorescence, like the VCF data (Figure 6C). In addition, there is no shift in the G(V) nor F(V) curves (Figures 7H, I). Thus, merely slowing pore transitions recapitulates most observed effects of ML277. The best simple fit for the effects of R-L3 occur by altering a different gating mechanism from that of ML277. Instead of slowing pore opening/closing, we decreased the deactivation rate of the main voltage sensor transition by 240% in our model. This increases the time constant of fluorescence deactivation by 224% and the time constant of current deactivation by 187% (Figures 6E–G). Furthermore, we found a hyperpolarizing shift in both F(V) and G(V) (Figures 6J, K). Finally, we increased current amplitude in our model by 24% to reflect experimental results. These simulations recapitulate most of R-L3s effects from our VCF data.

As the modeling required increases in current amplitude to reproduce the effect, one possibility was an effect on channel trafficking. To address this, we utilized a fluorescence-activated cell sorting assay to conveniently measure BBS-KCNQ1-YFP channel surface expression and total pools. As previously described (Kanner et al., 2017; Kanner et al., 2018; Kanner et al., 2020), a 13-residue high-affinity bungarotoxin-binding site (BBS) was introduced into the extracellular S1–S2 loop of KCNQ1 as an extracellular epitope tag to enable efficient detection of channel surface expression with Alexa Fluor 647-conjugated bungarotoxin (BTX_647_). YFP tag was fused to the C-terminus of KCNQ1 to enable efficient fluorescence detection of total KCNQ1 channel expression (Figure 7). Three types of control experiments were performed: untransfected, BBS-KCNQ1, and KCNQ1-YFP constructs. Figure 7 left showed that the system works with robust fluorescence signals for total (YFP) and surface (BTX_647_) channel pools. In the BBS-KCNQ1-YFP group, 1 μM ML277 or 1 μM R-L3 was added into the wells for 10 min prior to the experiments. Double-parallel control (without drugs) was performed. The total channel expression (YFP) or surface expression (BTX_647_) showed little or no change in cells treated with 1 μM ML277 or 1 μM R-L3 compared to double-parallel control (Figure 7, compare left and right panels), suggesting that ML277 or R-L3 had no effect on the trafficking of KCNQ1 channel, even though currents of KCNQ1 had increased significantly.

## Discussion

Both ML277 and R-L3 are potent activators of KCNQ1 channel and can greatly enhance current amplitude in the sub-micromolar to micromolar ranges. They also significantly slow channel deactivation as manifested by the “flatter” tail current recorded at repolarizing voltages (Yu et al., 2013). Both compounds share an interesting but undesired common feature. That is, channel sensitivities to these molecules are determined by the stoichiometry between KCNQ1 and KCNE1. Beneath the surface of these similarities, however, we now demonstrate the subtle differences in underlying mechanisms of these two activators, especially in their abilities to affect voltage sensor movements.

As other voltage-gated potassium channels, the transmembrane domain of KCNQ1 consists of two functional modules, a voltage sensor composed of the first four transmembrane helices (S1–4) and a pore domain composed of S5, pore loop, and S6. Our results suggest that a pore-dependent, voltage sensor–independent mechanism underlies ML277s current-enhancing effect. This is evidenced by the following findings. First, chimera C1 constructed using KCNQ1 pore domain and voltage-sensing domain of KCNQ2, which is insensitive to ML277, was able to respond to ML277 with increased current amplitude and slowed deactivation, suggesting that the voltage-sensing domain of KCNQ1 is not necessary for ML277 action and that the drug sites are likely confined within the pore domain. Second, we subsequently identified, in the KCNQ1 pore domain, eight residues (L266, Y267, G269, and G272 on S5 and V334, F335, F339, and F340 on S6) necessary for ML277 effects. Mutations of each of these eight residues rendered the channel irresponsive to ML277. Third, we measured both conductance–voltage G(V) and fluorescence–voltage F(V) relationships of KCNQ1 and did not observe changes in either G(V) or F(V) relationships as a result of ML277 application. Last, the mild effect of ML277 to slow fluorescence deactivation, according to our kinetics modeling, is likely due to a retrograde effect secondary to ML277s direct effect on KCNQ1 pore. We thus conclude that ML277 does not seem to affect voltage sensor directly. In contrast, R-L3 not only shifts G(V) and F(V) to negative potentials but also prominently slows deactivating movement of KCNQ1 voltage sensor. We also observed that several mutations in the putative hydrophobic pocket abolished the effect of R-L3 on current amplitude but not on deactivation kinetics, highly suggestive of a separate mechanism that may directly affect voltage sensor movement.

Unlike their differential effects on voltage sensor, ML277 and R-L3 seem to share a similar binding pocket on KCNQ1. Based on our hypothesis, ML277 binds in a hydrophobic pocket in the mid-section of the S5–S6 pore domain. R-L3 binding shares some common residues within the same pocket. The pocket is exposed to lipids; thus, both compounds can access their targets readily via the cell membrane. The side chains of the necessary residues are facing lipid instead of the ion permeation pathway. This potentially allows ML277 and R-L3 to modify the pore structure without blocking it. Without assaying the physical interaction between the channel and the molecule, we cannot discern whether these residues are actual drug-binding sites or simply sites needed for transducing conformational changes of pore structure upon drug binding. Nevertheless, it is reasonable to conclude that the integrity of the hydrophobic pocket formed by these residues is absolutely necessary for ML277 action and for R-L3 to a lesser degree.

The identified critical residues are clustered in the interface between two neighboring channel subunits. The inter-subunit location of the putative hydrophobic pocket provides a likely explanation to the interesting phenomenon that the current-enhancing effect of ML277 and R-L3 is dependent on the stoichiometry between KCNQ1 and KCNE1. That is, the more KCNE1 subunits in the channel, the less efficient it is for ML277 or R-L3 to enhance current amplitude. We have previously reported that some residues near the extracellular ends of KCNQ1 S6 and KCNE1, when mutated to cysteine, can spontaneously form disulfide bond and be cross-linked, suggesting that KCNE1 is likely located in the interfaces between two KCNQ1 subunits and is in close proximity with S6 of KCNQ1 (Chung et al., 2009). The occupancy of KCNE1 in this region, thus, poses a steric hindrance, limiting the accessibility of these two drugs to their inter-subunit hydrophobic binding pocket, thus preventing them from modifying the channel pore conformation.

The effects of ML277 on KCNQ1 are reminiscent of KCNE1 in that both greatly increase current amplitude. This suggests that both ML277 and KCNE1 may interact with key residues in the same pore region and have similar impact on channel pore conformation. Indeed, the triplet residues S338, F339, and F340 in KCNQ1 S6 are known to interact specifically with different KCNE subunits and are critical for KCNQ1 channel conformational changes (Melman et al., 2004; Panaghie et al., 2006). Both F339 and F340 are implicated in our study, which are necessary for ML277 actions. In a separate report, Nakajo et al. (2011) demonstrated that mutations of G272 and V334, which were shown necessary for ML277 effect, diminished the effect of KCNE1 on KCNQ1 G(V) relationship, while other aspects of KCNE1 modulation such as current augmentation were unaffected. These findings together with ours suggest that the mid-section of the KCNQ1 pore domain is critical for channel modulation. It is important to caution that ML277 does not fully mimic KCNE1, probably because KCNE1 is a much larger molecule than ML277, and makes extensive contacts with KCNQ1 not only in the pore domain but also in the voltage-sensing domain (Nakajo and Kubo, 2007; Nakajo and Kubo, 2011) and extracellular regions (Xu et al., 2008; Chung et al., 2009).

The mid-section of S5 and S6 is also the target of other channel activators. Interactions of small molecules with key residues in the pore domains of KCNQ1 and other voltage-gated potassium channels often result in modified channel biophysical properties, including increased current amplitude and altered gating behaviors. In the current study, we showed that both ML277 and R-L3 share common sites to target KCNQ1. A previous report showed that alanine mutations of certain residues in the same region on KCNQ1 greatly decrease the sensitivity to R-L3. These residues include Y267, I268, L271, and G272 on S5 and F335 and I337 on S6 (Seebohm et al., 2003b). In our control experiments shown in Figure 4, however, we did not observe mutation to KCNQ2 amino acids at G272 and F335 abolishing the effect of R-L3. Nevertheless, this shows that drugs targeting the mid-section of S5 and S6 may result in the upregulation of KCNQ1 function. In KCNQ3, a subunit of the neuronal M channel, W265, T271 on S5, and L338 on S6 are targets of retigabine, an anticonvulsant and an M channel activator (Lange et al., 2009). These residues are equivalent to ML277 sites L266, G272, and V334 on KCNQ1, respectively, based on the amino acid sequence alignment.

In a recent study Xu et al. adopted a different strategy to dissect ML277 sites on KCNQ1. Using molecular dynamics and small-molecule docking simulation, they identified two groups of residues necessary for ML277 effects, one in the voltage-sensing domain (R195, R243, and R249) and the other in the pore domain (F272, F332, F335, and F339) (Xu et al., 2015). While mutations of these residues did reduce the increase in KCNQ1 current amplitude by ML277 to variable degrees (albeit smaller than the mutants we identified), all but two still responded to ML277 (e.g., R243Q and F339A showed a greater than 50% increase in current amplitude) and showed the characteristic slower deactivation kinetics. In contrast, mutations of all residues we identified completely abolished both increase in current amplitude and change in deactivation kinetics.

Our modeling results allowed us to examine the gating mechanisms affected by these drugs and provide a further indirect conformation of the binding locations. For ML277, the gating effects are entirely reproduced with voltage-independent slowing of pore opening and closing, consistent with a drug binding in the pore region. For RL-3, the gating effects were best fit by slow deactivation kinetics, suggesting some differences in binding wherein it influenced the voltage-sensing domain relaxation. This effect on the VSD could be due to direct interaction with that domain or an allosteric effect from a disturbance at the pore. From the flow cytometry results, we observed that the changes in current amplitude are not trafficking-dependent, which means there is likely a change in open-state conductance or a reduction in channel inactivation.

To summarize, we described a putative hydrophobic pocket in the KCNQ1 pore domain critical for the actions of channel activators ML277 and R-L3. We demonstrated that both compounds share common residues in the pocket necessary for their effect. In future work, we plan to investigate in a structural analysis whether the location of these residues is near the binding interface between KCNQ1 and key beta subunits. While ML277 directly affects the channel pore and is likely voltage sensor–independent, R-L3 directly modifies voltage sensor movement in addition to its effect on the pore.

## Data Availability

The raw data supporting the conclusions of this article will be made available by the authors, without undue reservation.
