# Supplementary material for: Two small-molecule activators share similar effector sites in the KCNQ1 channel pore but have distinct effects on voltage sensor movements
Source: Front Physiol. 2022 Jul 25;13:903050. doi: 10.3389/fphys.2022.903050 (PMC9359618; doi:10.3389/fphys.2022.903050)
Supplement: Supplementary file 1 [file DataSheet1.PDF]

Computational modeling

The model of channel gating use in this study arises from previous work[1]). Briefly the model consists of 12 states; 6 closed and 6 open, and two voltage sensor movements consistent with the growing knowledge of KCNQ1 gating features. The open states included states from different conformations of voltage sensing domains(O_0-O_4) as well as a concerted final open state referred to in some literature as the activated open state(O_5)[2]. Figure 1 shows the structure of the 12 state model and Table 1 shows the constants used for the simulations. The highlighted numbers represent changes from no-drug model.

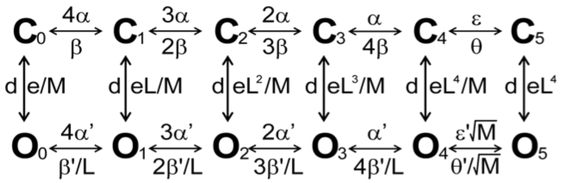

$$\alpha = c1 \cdot \exp(c2 \cdot (V_m - V_{half})) \qquad \alpha' = c4 \cdot \alpha$$
$$\beta = c1 \cdot \exp(-c3 \cdot (V_m - V_{half})) \qquad \beta' = c4 \cdot \beta$$
$$\varepsilon = c5 \cdot \exp(c6 \cdot (V_m - V_{half2})) \qquad \varepsilon' = c4 \cdot \varepsilon$$
$$\theta = c5 \cdot \exp(-c7 \cdot (V_m - V_{half2})) \qquad \theta' = c4 \cdot \theta$$

Figure S1: Schematic of gating scheme for KCNQ1 along with equations for values that are not strict constants

	KCNQ1	*ML277*	*R-L3*	*Units*
fl	0.8559	0.8559	0.8559	
fmax	1	1.95	1.24	
C1	1.079	1.079	0.5681	s^{-1}
C2	0.02977	0.02977	0.02977	mV^{-1}
C3	0.027	0.027	0.027	mV^{-1}
C4	0.9659	0.9659	0.9659	s^{-1}
d	34.03	2.127	34.03	s^{-1}
e	5.533	0.3458	5.533	s^{-1}
vHalf	-30.56	-30.56	-52.12	mV
L	1.833	1.833	1.833	
C5	1.187	1.187	1.187	s^{-1}
C6	0.01413	0.01413	0.01413	mV^{-1}
C7	0.01354	0.01354	0.01354	mV^{-1}
VHalf2	98.11	98.11	98.11	mV
M	1.399	1.399	1.399	

Table S1: Values for parameters used in simulations

BTX trafficking control experiments

For the flow cytometry experiments, three types of controls were performed- untransfected, BBS-KCNQ1 and KCNQ1-YFP constructs. These data are shown in Supplemental Figure S2.

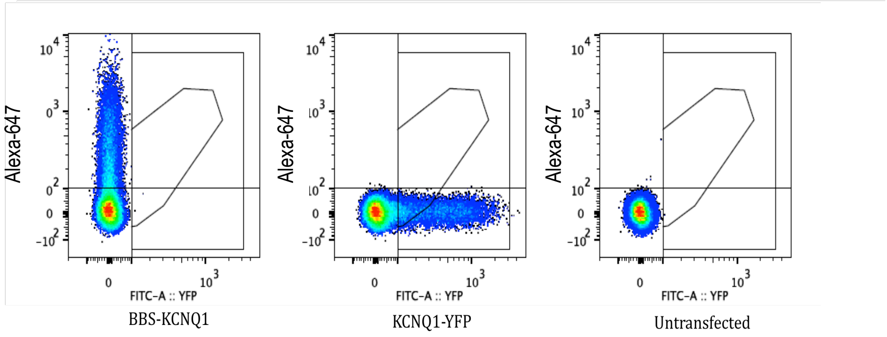

Figure S2: Control flow cytometry results for BBS-KCNQ1 alone(left), KCNQ1-YFP alone (middle), and untransfected show the system works as designed.

	peak current	deactivation
**WT**	119±8%	542±75%
**L266W**	93±3%	395±61%
**Y267A**	1±1%	-5±5%
**G269L**	12±5%	216±33%
**G272C**	99±10%	297±50%
**V334L**	78±4%	385±23%
**F335I**	41±4%	258±30%
**F339I**	23±5%	111±6%
**F340A**	-4±4%	298±25%

Table S2. R-L3 induced current increase (%) and Deactivation kinetics in all ML277 insensitive mutants

References

1. Peng, G., Barro-Soria, R. Sampson, K.J., Larsson, H.P. and Kass R.S. Gating Mechanisms underlying deactivation slowing by two KCNQ1 atrial fibrillation mutations. Sci Reports. 2017; 7:45911

2. Hou, P., Eldstrom, J., Shi, J., Zhong, L., McFarland, K, Gao, Y., Fedida, D and Cui, J. Inactivation of KCNQ1 potassium channels reveals dynamic coupleinf between voltage sensing and pore opening. *Nat Commun*. 2017; 8: 1730.
